# Application of Dragonnet and Conformal Inference for Estimating Individualized Treatment Effects for Personalized Stroke Prevention: Retrospective Cohort Study

**DOI:** 10.2196/50627

**Published:** 2025-01-08

**Authors:** Sermkiat Lolak, John Attia, Gareth J McKay, Ammarin Thakkinstian

**Affiliations:** 1 Department of Clinical Epidemiology and Biostatistics, Faculty of Medicine, Ramathibodi Hospital, Mahidol University, 4th Floor, Sukho Place Building, 218/11 Sukhothai Road, Suan Chitlada, Dusit, 10300, Thailand, 66 955073078; 2 Centre for Clinical Epidemiology and Biostatistics, School of Medicine and Public Health, Hunter Medical Research Institute, University of Newcastle, New Lambton, New South Wales, Australia; 3 Centre for Public Health, School of Medicine, Dentistry and Biomedical Sciences, Queen’s University Belfast, Belfast, United Kingdom; 4 Department of Clinical Epidemiology and Biostatistics, Faculty of Medicine, Ramathibodi Hospital, Mahidol University, Bangkok, Thailand

**Keywords:** stroke, causal effect, ITE, individual treatment effect, Dragonnet, conformal inference, mortality, hospital records, hypertension, risk factor, diabetes, dyslipidemia, atrial fibrillation, machine learning, treatment

## Abstract

**Background:**

Stroke is a major cause of death and disability worldwide. Identifying individuals who would benefit most from preventative interventions, such as antiplatelet therapy, is critical for personalized stroke prevention. However, traditional methods for estimating treatment effects often focus on the average effect across a population and do not account for individual variations in risk and treatment response.

**Objective:**

This study aimed to estimate the individualized treatment effects (ITEs) for stroke prevention using a novel combination of Dragonnet, a causal neural network, and conformal inference. The study also aimed to determine and validate the causal effects of known stroke risk factors—hypertension (HT), diabetes mellitus (DM), dyslipidemia (DLP), and atrial fibrillation (AF)—using both a conventional causal model and machine learning models.

**Methods:**

A retrospective cohort study was conducted using data from 275,247 high-risk patients treated at Ramathibodi Hospital, Thailand, between 2010 and 2020. Patients aged >18 years with HT, DM, DLP, or AF were eligible. The main outcome was ischemic or hemorrhagic stroke, identified using *International Classification of Diseases, 10th Revision* (*ICD-10*) codes. Causal effects of the risk factors were estimated using a range of methods, including: (1) propensity score–based methods, such as stratified propensity scores, inverse probability weighting, and doubly robust estimation; (2) structural causal models; (3) double machine learning; and (4) Dragonnet, a causal neural network, which was used together with weighted split-conformal quantile regression to estimate ITEs.

**Results:**

AF, HT, and DM were identified as significant stroke risk factors. Average causal risk effect estimates for these risk factors ranged from 0.075 to 0.097 for AF, 0.017 to 0.025 for HT, and 0.006 to 0.010 for DM, depending on the method used. Dragonnet yielded causal risk ratios of 4.56 for AF, 2.44 for HT, and 1.41 for DM, which is comparable to other causal models and the standard epidemiological case-control study. Mean ITE analysis indicated that several patients with DM or DM with HT, who were not receiving antiplatelet treatment at the time of data collection, showed reductions in total risk of −0.015 and −0.016, respectively.

**Conclusions:**

This study provides a comprehensive evaluation of stroke risk factors and demonstrates the feasibility of using Dragonnet and conformal inference to estimate ITEs of antiplatelet therapy for stroke prevention. The mean ITE analysis suggested that those with DM or DM with HT, who were not receiving antiplatelet treatment at the time of data collection, could potentially benefit from this therapy. The findings highlight the potential of these advanced techniques to inform personalized treatment strategies for stroke, enabling clinicians to identify individuals who are most likely to benefit from specific interventions.

## Introduction

Stroke is a leading cause of death and disability, presenting both personal and economic burdens [1]. Astonishingly, many epidemiological studies have identified important risk factors of stroke occurrence, especially through the use of cohort studies [2], and randomized controlled trials (RCTs) have identified the impact of treating these risk factors. While RCTs control for confounding factors through study design, cohort studies attempt to address these factors using statistical methods. However, the possibility of residual confounding remains, highlighting the need for improved analysis approaches [3].

Frameworks of causal effect have largely been confined to Pearl’s [4] structural causal models (SCMs) and Rubin’s [5] potential outcome models (POMs) [6]. SCMs evaluate causal relationships between variables using a directed acyclic graph defined by a set of structural equations, which consider the influence of each variable by its parents, or causes, along with its probability distribution. In addition, SCMs can also assess the effect of interventions by estimating how changing one unit of treatment (or risk) leads to a change in outcome [7]. Conversely, POMs focus on the concept of counterfactuals, specifically what would have happened if an individual had been exposed to a different treatment or risk [8]. Consequently, this approach estimates 2 potential outcomes (POs) for each individual: if the individual had received the treatment and if they had not. Subsequently, Rosenbaum and Rubin [9] developed propensity scores to reflect the probability of an individual being assigned to a certain treatment group. Therefore, these estimates are only considered valid if the 2 specific conditions—strong ignorability and positivity—are met. Statistical methods have been developed based on POMs and propensity scores, including matching [10], stratified propensity score (SPS) [11], inverse probability weighting (IPW) [12,13], and doubly robust estimation (DRE) [14-16]. Recently, nonconventional statistical models such as double machine learning (DML), meta-learners, and neural networks have also been developed to estimate unbiased causal effects without requiring strong underlying assumptions [14]. Causal neural networks (NNs), including TARNet and Dragonnet, learn by sharing input data to estimate both factual and counterfactual outcomes. This approach is currently an active area of research [17-19]. Dragonnet also uses “learned data” to predict propensity scores by tradeoff with prediction quality, which yields better average treatment effect (ATE) estimates [18].

Current causal modeling has shifted its focus from the ATE, which measures the treatment effect averaged across the entire study population, to the conditional average treatment effect (CATE), which assesses the ATE conditional on particular variables, such as sex, age, and other covariates. More recently, the focus has further evolved to the individualized treatment effect (ITE), which estimates the treatment effect for a particular individual. CATE has inherent variability depending on which covariate the model is conditioned on [20]. However, estimating ITEs is challenging because it requires making assumptions about the underlying individual data-generating process and the model used to estimate the ITEs [17]. A statistical technique called conformal inference may appropriately estimate the confidence intervals of ITEs by accounting for the uncertainty in their estimation. Despite being a novel technique, it has shown promise [20]. Conformal inference uses nonconformity scores that measure the degree of disagreement between the estimated and observed outcomes, to provide a confidence interval or a precision of estimation [21-23]. Therefore, we conducted this study to estimate the CATE of stroke occurrence based on real-world clinical data using Dragonnet NN models. Additionally, ITE was estimated to identify individuals at high risk of stroke who may benefit from lowering risk factors by combining the strengths of Dragonnet and conformal inference approaches. To the best of our knowledge, no prior studies have employed these methods in combination to estimate causal effects in a clinical setting.

## Methods

### Overview

The study population included a retrospective cohort of patients who were at high risk for stroke and had been treated and followed up at Ramathibodi Hospital, Thailand, between 2010 and 2020. Hospital records and the *International Classification of Diseases, 10th Revision* (*ICD-10*) classification system were used to identify patients. Patients were eligible if they were aged >18 years and had one or more of the following conditions: hypertension (HT; *ICD-10* code I10-I16), diabetes mellitus (DM; *ICD-10* code E08-E13), dyslipidemia (DLP; *ICD-10* code E78), and atrial fibrillation (AF; *ICD-10* code I48). Patients were excluded if they had a stroke on their first visit or only had one visit during the study period. The main outcome measured in the study was the occurrence of ischemic or hemorrhagic stroke, which was identified using the *ICD-10* codes I63 and I61, respectively.

**Figure 1.**
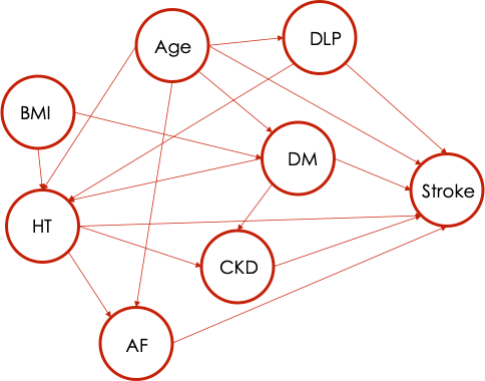
Causal diagram of patients at risk of stroke occurrence. AF: atrial fibrillation; CKD: chronic kidney disease; DLP: dyslipidemia; DM: diabetes mellitus; HT: hypertension.

Patients were followed up from their index date (i.e., the date they were identified as high-risk patients) until they progressed to stroke, were lost to follow-up, or were stroke-free at the end of the study (December 31, 2020). Patients who were lost to follow-up or stroke-free at the end of the study period were censored on their last visit date or at the end of the study. A causal diagram was constructed (Figure 1), and potential predictors of stroke were collected, including age, sex, BMI, chronic kidney disease (CKD), AF, HT, DM, and DLP. HT, AF, and DM were considered as mediators, whereas the remaining variables were covariates in the models. A software library called DoWhy, now incorporated into PyWhy (Python Software Foundation), was used to construct models for stratification, IPW, DRE, and DML [24]. Parameters of all estimators were set by default in the DoWhy package. The number of strata in the stratification method was automatically determined [25]. The weighting scheme in IPW was set to default inverse propensity score. For DRE, the regression and propensity models were specified as lasso and logistic regression, respectively. For DML, linear and nonlinear cross-fitted models were applied to the outcome model (lasso and Extreme Gradient Boosting [XGBoost]), propensity model (logistic regression and XGBoost), and final model (linear regression and lasso). Estimands of each risk pathway were defined by PyWhy from the input causal graph. Graphical causal model–based inferences from the DoWhy library were used for medication analysis to quantify the causal effects of direct and indirect pathways, termed natural direct effect (NDE) and natural indirect effect (NIE), respectively [4,26]. NDE $\left(Y_{1,M(0)}^{x}-Y_{0,M(0)}^{x}\right)$ refers to the change in the outcome of an individual when they are exposed to a specific treatment $Y_{1}$, compared to another treatment $Y_{0}$, while keeping the mediator variable constant at the baseline value or reference treatment *M(0*). In contrast, NIE $\left(Y_{1,M(1)}^{x}-Y_{1,M(0)}^{x}\right)$ refers to the difference between the counterfactual outcome value when treatment $Y_{1}$ is fixed and the mediator assumes a certain value at a particular treatment *M(1*) and the counterfactual outcome value when the mediator assumes the same value at the baseline *M(0*) [27].

**Figure 2.**
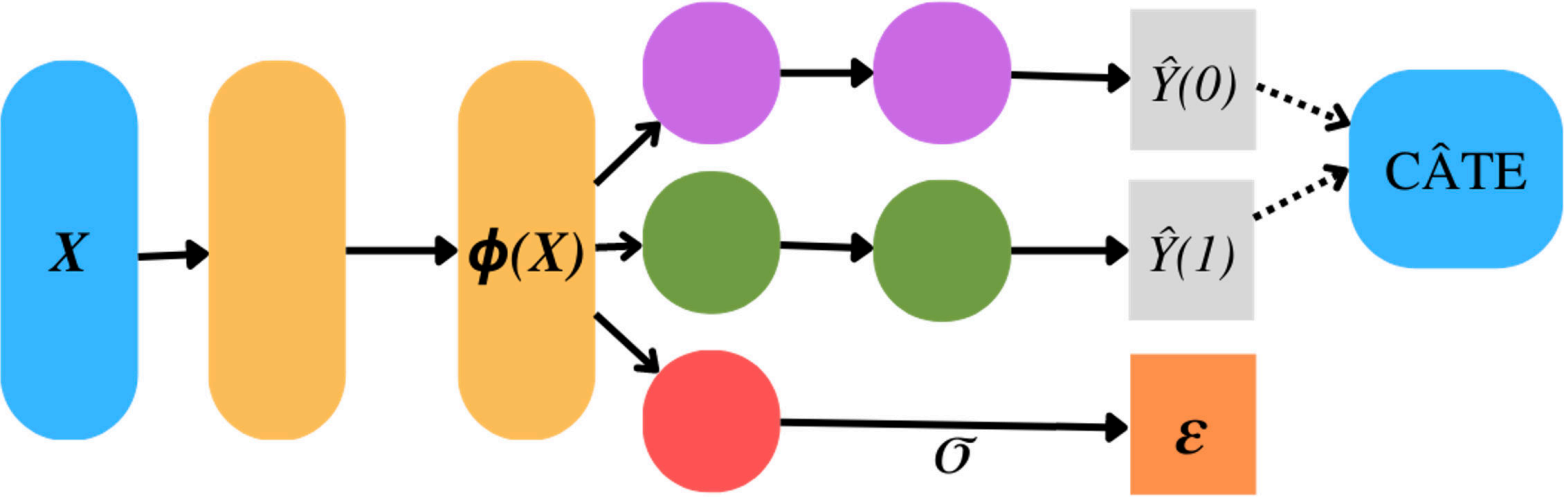
Dragonnet architecture. *X* is the covariates, *ɸ(X)* is a learned representation of *X*. *Ŷ(1)* is the predicted outcome of the treatment (risked) group. *Ŷ(0)* is the predicted outcome of the control group. *ε* is the estimated propensity score. CÂTE is the conditional average treatment effect computed by *Ŷ(1)–Ŷ(0)*.

The Dragonnet NN was used to estimate PO and propensity scores. The architecture of Dragonnet was based on previous work (Figure 2) [18]. It employs a deep net to create a representation layer *ɸ(X*) ∈ ℝᴾ, which is used to forecast outcomes for both the treatment *Ŷ(1*) and control groups *Ŷ(0*). It utilizes 2 hidden layers for each outcome model while a basic fully connected layer with a sigmoid function is used for the propensity score (𝜺). CATE was estimated by subtracting treatment (risk) and control PO for each risk factor $\left(Y_{1}^{x}-Y_{0}^{x}|Z\right)$ and risk ratios were estimated by division of PO ($\frac{Y_{1}^{x}}{Y_{0}^{x}}|Z$); *Y₁* is the PO for the risk group, *Y₀* is the PO for the control group, *x* is an interested factor, and *Z* are other covariates.

To accurately estimate the ITE, it is mandatory for the conditional independence assumption to hold, especially considering the unequal distribution of covariates between factual and counterfactual outcomes of the treatment and control groups, commonly known as covariate shift. To address this challenge, we employed a nested method of weighted split-conformal quantile regression (CQR) to estimate the ITE [20,23] by incorporating antiplatelet medications as a treatment for stroke prevention. POs were estimated using quantile loss setting α at .05. The dataset was split evenly into training and evaluation sets; Multimedia Appendix 1 shows the entire algorithm. All risk factors and covariates were similar between models, considering antiplatelet medication as a treatment and stratified by risk factor $(Y_{antiplatelets=1}^{x}-Y_{antiplatelets=0}^{x}|Z),$ with *x* representing the risk factors of interest (i.e., HT, DM, and DLP) and Z representing other covariates. AF was not included as a stratum for the estimation of ITE in this example since it is not an indication for the prescription of antiplatelet therapy, but it remained a covariate.

### Ethical Considerations

The data were anonymized to ensure confidentiality and privacy protection. This study was approved by the Human Research Ethics Committee, Faculty of Medicine Ramathibodi Hospital, Mahidol University (COA. MURA2021/255). The committee waived the need to obtain consent for the collection, analysis, and publication of the retrospectively obtained and anonymized data for this noninterventional study.

## Results

A total of 275,247 high-risk patients were included in the cohort. Among them, 9659 patients developed stroke, resulting in an incidence of 3.5% (95% CI 3.4-3.6). The follow-up rate for the study population was 80% (7752/9659).

Baseline demographic and risk factors were compared between 9659 stroke patients and 265,588 nonstroke patients (Multimedia Appendix 2). Stroke patients had a mean age of 64.7 years and were more likely to be male. Stratification by risk indicated that 13% of AF patients, 4% of HT patients, 4% of DM patients, and 4% of DLP patients experienced stroke in contrast to only 2% of non-AF patients, 1% of non-HT patients, 3% of non-DM patients, and 3% of non-DLP patients, who developed stroke.

Causal effects of mediators including HT, DM, CKD, and AF on stroke were estimated based on the causal diagram in Figure 1. The estimands report as probability of stroke given the risk factors, *P*(*Stroke* | *risk factors*), are as follows: *P*(*Stroke* | *HT, age, DM, DLP*) for HT; *P*(*Stroke* | *AF, age, HT*) for AF*; P*(*Stroke* | *age, DLP*) for DLP; and *P*(*Stroke* | *age, DM, BMI*) for DM (Multimedia Appendix 3). For the POM approach, the SPS estimator showed AF as the highest risk of stroke, followed by HT, DM, and DLP with risk estimates of 0.084 (95% CI 0.079-0.088), 0.019 (95% CI 0.015-0.020), 0.010 (95% CI 0.008-0.010), and 0.0015 (95% CI −0.0002 to 0.0027), respectively. IPW yielded similar, albeit slightly higher, corresponding risks of 0.092 (95% CI 0.089-0.096), 0.024 (95% CI 0.022-0.025), 0.010 (95% CI 0.008-0.010), and 0.001 (95% CI −0.0005 to 0.0025), respectively. Comparable results were observed in the DRE analysis, with a similar trend of risk effect estimates of 0.082 (95% CI 0.0849-0.0871), 0.025 (95% CI 0.0243-0.0257), 0.008 (95% CI 0.0057-0.0063), and 0.0006 (95% CI 0.0001-0.0011), respectively.

The SCM estimation also yielded similar trends to the POM approach, in which the risk of stroke was 0.096 (95% CI 0.0948-0.0972), 0.021 (95% CI 0.0204-0.0216), 0.007 (95% CI 0.0067-0.0073), and 0.0005 (95% CI 0.0004-0.0006) for AF, HT, DM, and DLP, respectively. Mediation analysis indicated the NDE of HT to be 0.020 (95% CI 0.019-0.021) and the NIE to be 0.0027 (95% CI 0.0025-0.0029). NDE and NIE for DM and DLP were both modest and consistent with the findings from other models. Figure 1 illustrates the pathways through which the mediators act: HT mediates through CKD and AF, DM mediates through HT and CKD, while DLP mediates through HT.

In the context of DML, the nonparametric model estimates were slightly smaller than those for the linear model, with risks of 0.086 (95% CI 0.0849-0.0871), 0.015 (95% CI 0.0145-0.0155), 0.006 (95% CI 0.0057-0.0063), and 0.0 (95% CI −0.0001 to 0.001) for AF, HT, DM, and DLP, respectively, whereas the corresponding linear model estimate risks were 0.097 (95% CI 0.096-0.098), 0.023 (95% CI 0.0223-0.0236), 0.009 (95% CI 0.0087-0.0093), and 0.002 (95% CI 0.0018-0.0022).

Dragonnet estimated the causal effects of AF, HT, DM, and DLP on stroke as 0.075 (95% CI 0.074-0.076), 0.017 (95% CI 0.0169-0.0170), 0.01 (95% CI 0.009-0.010), and −0.002 (95% CI −0.0022 to 0.0021), with causal ratios of 4.56 (95% CI 4.56-4.57), 2.44 (95% CI 2.41-2.46), 1.41 (95% CI 1.21-1.60), and 0.856 (95% CI 0.855-0.858), respectively. The odds ratios from the logistic regression models were respectively 3.34 (95% CI 2.68-3.75), 2.56 (95% CI 2.33-2.80), 1.16 (95% CI 1.05-1.30), and 1.00 (95% CI 0.8-1.4). Details are provided in Multimedia Appendix 3 for comparison.

**Figure 3.**
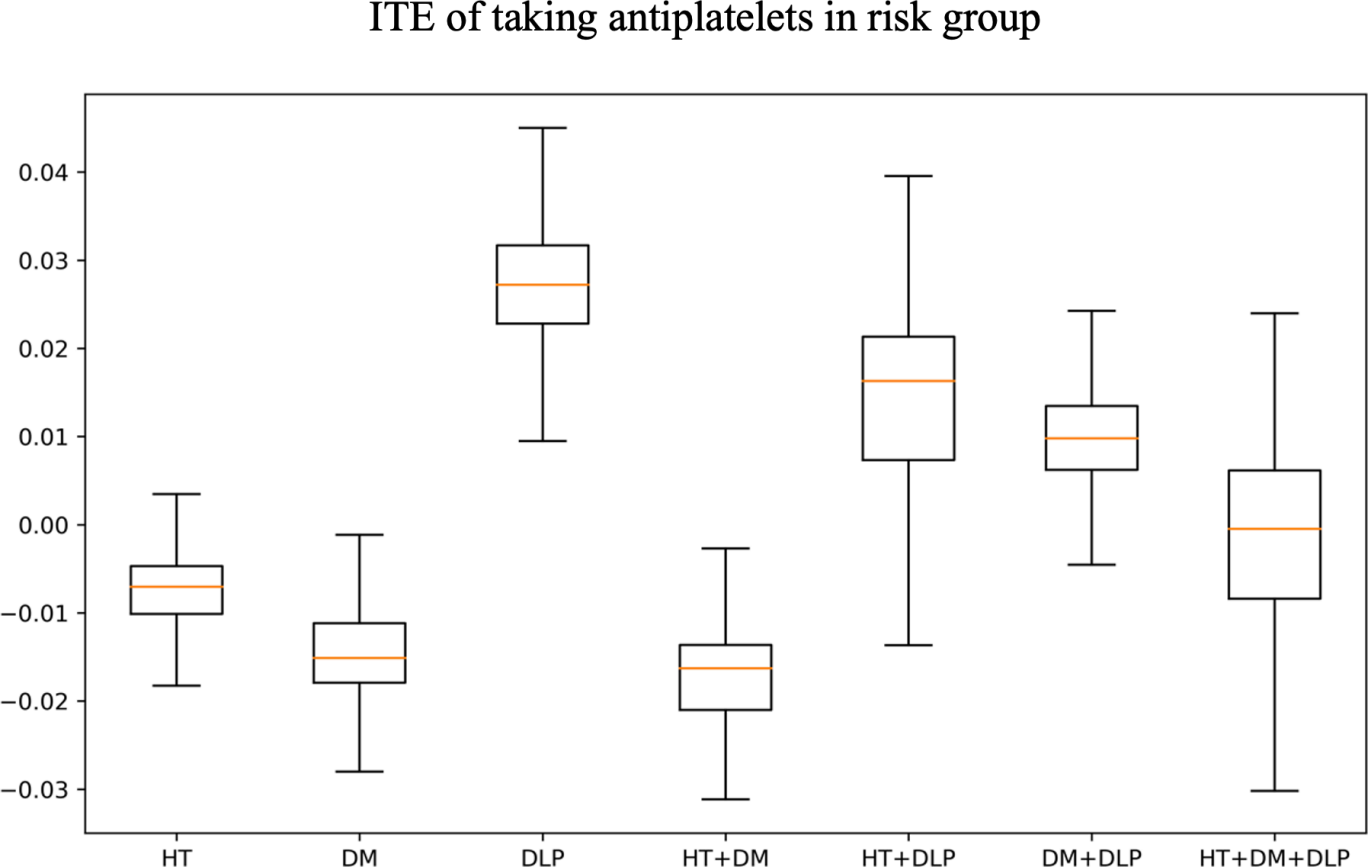
Box plot representing the mean individual treatment effect for patients with different risk factors who had not been taking antiplatelet medication, illustrating the potential impact on stroke risk reduction if they had received antiplatelet therapy. DLP: dyslipidemia; DM: diabetes mellitus; HT: hypertension; ITE: individual treatment effect.

The influence of risk reduction for individual patients who did not receive antiplatelet therapy, had they been given the medication (counterfactuals of nontreatment ITEs), was examined using weighted split-CQR. As shown in Multimedia Appendix 4, three of the samples (3/50, 6%) appear to have potentially benefited from antiplatelet treatment, indicating that a considerable number of patients might have experienced a positive impact on their stroke risk reduction had they received the medication. The mean ITEs indicated that several patients with DM or DM with HT were not currently receiving antiplatelet treatment and would be more likely to benefit if they had received it, with reduction of total risk as −0.015 (IQR −0.011 to −0.018) and −0.016 (IQR −0.015 to 0.022) among each group, respectively (Figure 3).

## Discussion

### Principal Findings

We estimated the causal influences of risk factors associated with stroke outcomes using multiple approaches that included SPS, IPW, DRE, SCM, and mediation analysis, in addition to DML and Dragonnet NNs. Our findings indicate strong positive causal effects associated with AF and HT on stroke development, with DM exerting a weaker effect. DLP, in contrast, had little effect. Furthermore, our analysis suggests that patients with both DM and HT not currently in receipt of antiplatelet treatments would be the most likely beneficiaries of antiplatelet therapy based on the mean ITEs.

The results from the different estimators generally demonstrated consistency, although there were slight variations in specific point estimates and confidence intervals varied slightly. The estimated causal effect derived from various methods using real-world observational data is comparable with standard cohort epidemiological studies using more traditional logistic regression approaches [28,29].

### Comparison to Prior Works

SPS is a widely used method that minimizes confounding bias by adjusting baseline covariates and confounding factors and estimating treatment effects by stratum. However, SPS is sensitive to the number of strata and features that affect both treatment and outcome (confounding factors), which can lead to bias in the causal effect estimate [30-33]. In addition, some strata may be sparsely populated, making the ATE hard to define and more prone to bias [34]. Rosenbaum and Rubin [9] originally proposed dividing the strata into 5 levels and then subsequently automatically splitting the strata until the balance in the numbers of treated and control observations was achieved [25].

IPW attempts to reduce confounding of the ATE by weighting the sample with the inverse propensity score and by balancing the distribution of the covariates between the treated and untreated groups [35], thereby avoiding the problem of data sparsity that may be present in SPS, particularly with small sample sizes. However, there is a reliance on the assumption that the propensity score model correctly captures all confounding factors, which, if incorrect, may bias the ATE. Additionally, IPW is more sensitive to the model and variable selection for estimating the propensity scores, with small differences in estimated propensity scores potentially leading to large differences in estimated causal effects [36]. Finally, IPW may imprecisely estimate treatment effects if a sample size is small, leading to a propensity score close to 0 or 1 [36,37].

DRE combines propensity score and outcome regression models [38], which can lead to improvements in the robustness of model specification by allowing one of the two treatment and outcome models to be miss-specified but still provide a consistent estimation [39]. The challenge is to validly model either the propensity score or the outcome model; it may be tempting to use modern machine learning approaches or nonparametric models in DRE, but this may lead to bias if the functions are too complex, leading to overfitting [40,41]. DML was developed to address the bias from regularization and overfitting in estimating the parameter of interest, which arises when naively inserting machine learning estimators into the estimation equation. This approach consists of two critical components: (1) the use of Neyman-orthogonal moments or scores to estimate the parameters and (2) the application of cross-fitting, which provides an efficient form of data-splitting. By using both elements, DML minimizes the impact of regularization bias and overfitting on parameter estimation; this also extends to nonparametric models [14].

Applying POMs (eg, SPS, IPW, DRE) relies heavily on the assumption that the treatment assignment is independent of the PO given the observed covariates, which is known as “unconfoundedness” or the conditional independence assumption. If this assumption does not hold, the estimated causal effect will be biased. In contrast, SCMs facilitate the modeling of complex relationships between multiple causes and effects in the presence of latent or unobserved variables [4,42]. In addition, SCMs can be considered as counterfactual predictions of interventions, which can be useful in applications such as causal inference in experimental or observational studies [43-46]. However, SCMs are limited by the assumption of independence between variables and may require conceptualized causal relationship mechanisms.

The benefit of using NNs to estimate causal effects is their flexibility and power to handle high-dimensional and complex data. Shalit et al [17] introduced TARNet by sharing information between the PO of treatment and control groups, which is different from the previous model that separated the training data. More recently, Dragonnet was developed by combining propensity scores with targeted regularization, resulting in more accurate inference [18]. Dragonnet is considered more robust with very low or high propensity scores but has several limitations including sensitivity to choice of architecture and hyperparameters, dealing with only a single set of features at a time, and difficulty of interpretation [18]. Despite some limitations, Dragonnet’s benefits surpass these drawbacks, making it an attractive approach for estimating causal effects in complex real-world data.

### Strengths and Limitations

A critical aspect of causal inference, particularly in estimating CATE, involves certain assumptions, notably ignorability and positivity. Strong ignorability necessitates the observation and adjustment for all confounding variables that influence both the treatment and the outcome, while positivity ensures that every patient has a nonzero probability of receiving each treatment. In our study, we believe these assumptions are reasonably satisfied. We included a comprehensive set of covariates, such as age, sex, BMI, chronic kidney disease, and relevant comorbidities (HT, DM, DLP, and AF), which are well-documented factors influencing stroke risk and treatment decisions. However, we acknowledge that there might be unmeasured confounders not captured in our dataset. Regarding the decision on antiplatelet drug administration, we utilized detailed patient records from Ramathibodi Hospital, ensuring a thorough assessment of factors influencing treatment. Nonetheless, we recognize the potential for residual confounding and the inherent limitations of observational data. Future studies could benefit from incorporating more granular clinical data and leveraging advanced causal discovery methods to further validate these assumptions.

Causal effects can vary between individuals, which necessitates the estimation of ITEs. Treatment effects can vary between individual patients; therefore, applying a single treatment effect as CATE to all individual patients is inappropriate [47,48] as some patients may gain more or less benefit from treatments. Thus, the estimation of ITE to identify at-risk patients most likely to benefit from treatment is a major goal for stratified and precision medicine approaches. Estimating ITEs requires larger sample sizes, as individual-level estimates are less precise than aggregate-level estimates [49]. A covariate shift may result from unobserved counterfactual data but this is minimized using a weighted split-CQR approach [23].

We believe that the clinical implications of our study are significant, as understanding the causal relationships and individual treatment effects of stroke risk factors can directly influence patient care by providing more precise and personalized risk assessments. Additionally, we can conduct reviews and quality assessments of current patients in the clinic to determine who should receive further treatment. These methods enable clinicians to identify high-risk patients who would benefit most from targeted interventions, like antiplatelet therapy, thereby optimizing treatment strategies and improving patient outcomes. The use of real-world data ensures that our findings apply to everyday clinical practice.

Our study has some limitations. First, we used real-world data rather than RCT data, thus some important covariates were not previously planned, measured, and collected as part of routine clinical evaluation and were therefore unavailable for ITE estimation. Second, we acknowledge the possibility of unmeasured confounders in the observational dataset. Future studies could benefit from incorporating more granular clinical data, such as detailed medication records, laboratory results, and lifestyle factors, to mitigate potential confounding. Third, the models used for estimating ITEs were trained and validated in only a single setting, thereby limiting their generalizability. Future research should focus on validating the models in diverse settings with different patient populations or hospitals. This external validation would help to determine whether the models’ predictive performance and the estimated ITEs hold true across various contexts.

### Conclusion

This study provides comprehensive causal estimates of AF, HT, DLP, and DM on stroke using various advanced statistical and machine learning methodologies. The consistent results across multiple analytical approaches and this study’s alignment with a standard cohort study reinforce the robustness of our findings. AF and HT emerged as significant risk factors for stroke, with DM showing a moderate effect, while DLP had minimal impact. Notably, the use of Dragonnet and conformal inference techniques allowed us to accurately estimate ITEs, highlighting that several high-risk patients who did not take antiplatelets at the time of data recorded, particularly those with DM or DM combined with HT, could potentially benefit from antiplatelet therapy. This suggests that personalized treatment strategies could be pivotal in reducing stroke risk among these patients.

The findings underscore the significance of individualized risk assessment and treatment personalization in clinical settings. Future research should focus on integrating these advanced causal inference models into routine clinical practice to enhance treatment outcomes for high-risk stroke patients. Additionally, the use of real-world data provides valuable insights but also presents challenges related to unmeasured confounding and data quality. Addressing these challenges in future studies will be crucial for advancing our understanding and improving stroke management strategies.

## Supplementary material

10.2196/50627 Multimedia Appendix 1 Nested approach for interval estimates of individual treatment effect algorithm. α=.05 to cover 95% confidence interval.

10.2196/50627 Multimedia Appendix 2 Descriptive analysis of features between stroke and nonstroke.

10.2196/50627 Multimedia Appendix 3 Estimated causal effect from estimators. Numbers indicate conditional average treatment (risk) effect (CATE) with 95% confidence interval. * Heart disease ** top quintile low-density lipoprotein (LDL).

10.2196/50627 Multimedia Appendix 4 Sample of 50 individual treatment effects with 95% confidence intervals and stroke risk reduction who had not received antiplatelet treatment, demonstrating the potential benefits had they been given the medication. In this plot, 3 of the samples (6%) demonstrate that a considerable number of patients could have experienced a positive impact on their stroke risk reduction had they received the antiplatelet treatment. The y-axis displays the treatment effect, while the x-axis represents each individual patient in the sample.

## Abbreviations

AF: atrial fibrillation
ATE: average treatment effect
CATE: conditional average treatment effect
CKD: chronic kidney disease
CQR: conformal quantile regression
DLP: dyslipidemia
DM: diabetes mellitus
DRE: doubly robust estimation
HT: hypertension
*ICD-10*: *International Classification of Diseases, 10th Revision*
IPW: inverse probability weighting
ITE: individualized treatment effect
NDE: natural direct effect
NIE: natural indirect effect
NN: neural network
PO: potential outcome
POM: potential outcome model
RCT: randomized controlled trial
SCM: structural causal model
SPS: stratified propensity score
XGBoost: Extreme Gradient Boosting
