# Supplementary material for: Application of Dragonnet and Conformal Inference for Estimating Individualized Treatment Effects for Personalized Stroke Prevention: Retrospective Cohort Study
Source: JMIR Cardio. 2025 Jan 8;9:e50627. doi: 10.2196/50627 (PMC11735012; doi:10.2196/50627)
Supplement: Multimedia Appendix 1 [file cardio-v9-e50627-s001.docx]

| **Algorithm:**  Nested approach for interval estimates individual treatment effect (ITE) |
| --- |
| **Input:** level α, data $Ƶ$ $=$ $\left( X_{i},Y_{i},T_{i} \right)_{i=1}^{n}$ |
| **Step I: data splitting.**   1. 1: Split the data into two folds $Ƶ_{tr}$and $Ƶ_{ca}$ 2. 2: Estimate propensity score $\hat{ⅇ}\left( x \right)$ on $Ƶ_{1}$ |
| **Step II: compute ITE**   1. Training the data with $Ƶ_{tr}$ by using quantile loss at $\propto/2$ and $1-(\propto/2)$ as $M_{lo}$, $M_{hi}$ 2. Training the data with $Ƶ_{tr}$by using mean square loss as $M_{e}$ 3. Predict data with $Ƶ_{ca}$using $M_{lo}$ for $\begin{aligned} {\hat{q}_{\alpha}}_{lo} \end{aligned}$ and $M_{hi}$ for ${\hat{q}_{\alpha}}_{hi}$ 4. Use predicted data to compute non-conformity score $\left\{ V_{i}=max\{\begin{aligned} {\hat{q}_{\alpha}}_{lo} \end{aligned} \right.\left( Xi \right)- Y_{i}, Y_{i}-\begin{aligned} {\hat{q}_{\alpha}}_{hi}\left( Xi \right)\} \end{aligned}$ 5. **for** *t* *in [0,1]* with *T = t*    1. *if t=0*; Compute weight by $w_{0}\left( x \right)=\hat{ⅇ}\left( x \right)/\left( 1- \hat{ⅇ}\left( x \right) \right)$ from predicted propensity score $(\hat{ⅇ})$ using $M_{e}$    2. *else*; Compute weight by $w_{1}\left( x \right)=(1-\hat{ⅇ}(x))/\hat{ⅇ}\left( x \right)$ from predicted propensity score $(\hat{ⅇ})$ using $M_{e}$    3. Compute the normalized weights $\hat{p}_{1}\left( x \right)= W_{i}/\left( \sum W_{i}+w_{t}\left( Xi \right) \right)$    4. Compute $\eta\left( x \right)$ as the $\left( 1-\alpha\right)$-th quantile of $[\hat{p}_{1}\left( x \right)V_{1}$, …, $\hat{p}_{n}\left( x \right)V_{n}]$    5. $\left[ \hat{Y}_{i}^{L}\left( t \right), \hat{Y}_{i}^{R}\left( t \right) \right]= [{\hat{q}_{\alpha}}_{lo}\left( x \right)-$ $\eta\left( x \right), \hat{q}_{\alpha_{hi}}(x)$ $+ \eta\left( x \right) ]$ 6. **end for** |
| **Output:** $\hat{C}_{ITE}\left( x \right)=[\hat{Y}_{i}^{R}\left( 1 \right)-\hat{Y}_{i}^{L}\left( 0 \right),\hat{Y}_{i}^{L}\left( 1 \right)-\hat{Y}_{i}^{R}\left( 0 \right)$] |
