# Supplementary material for: Application of Dragonnet and Conformal Inference for Estimating Individualized Treatment Effects for Personalized Stroke Prevention: Retrospective Cohort Study
Source: JMIR Cardio. 2025 Jan 8;9:e50627. doi: 10.2196/50627 (PMC11735012; doi:10.2196/50627)
Supplement: Multimedia Appendix 2 [file cardio-v9-e50627-s002.docx]

Table 1: Descriptive analysis of features between stroke and non-stroke

| Variable | Stroke (N=9659) | Non-Stroke (N=265588) | P-value |
| --- | --- | --- | --- |
| Age, years (Mean ± SD) | 64.7 ± 13 | 58 ± 14.1 | <0.001 |
| Male, n (%) | 5107 (53%) | 101700 (38.3%) | <0.001 |
| Body Mass Index, kg/m² (Mean ± SD) | 25.2 ± 4.4 | 25.5 ± 4.8 | <0.001 |
| Atrial Fibrillation, n (%) | 817 (8.5%) | 25454 (9.6%) | <0.001 |
| Hypertension, n (%) | 4202 (43.5%) | 94634 (35.6%) | <0.001 |
| Diabetes Mellitus, n (%) | 4012 (41.5%) | 99636 (37.5%) | <0.001 |
| Dyslipidemia, n (%) | 5067 (52.5%) | 112870 (42.5%) | 0.7 |
| Chronic Kidney Disease, n (%) | 7659 (79.3%) | 230935 (87%) | <0.001 |
| Antiplatelets, n (%) | 6791 (70.3%) | 217387 (81.9%) | <0.001 |

Table 2: Estimated causal effect from estimators.

| Column Head | Hypertension | Diabetes | Atrial Fibrillation | Dyslipidemia |
| --- | --- | --- | --- | --- |
| **Conventional estimator** |  |  |  |  |
| Stratification | 0.019 [0.015,0.020] | 0.010 [0.008,0.010] | 0.084 [0.079,0.088] | 0.0015 [-0.0002, 0.0027] |
| Inverse Probability Weighting | 0.024 [0.022,0.025] | 0.010 [0.008,0.010] | 0.092 [0.089,0.096] | 0.001 [-0.0005, 0.0025] |
|  |  |  |  |  |
| Doubly Robust Estimation | 0.025 [0.0243,0.0257] | 0.008 [0.0057,0.0063] | 0.082 [0.0849,0.0871] | 0.0006  [-0.0001,0.0001] |
|  |  |  |  |  |
|  |  |  |  |  |
| SCM | 0.021 [0.0204,0.0216] | 0.007 [0.0067,0.0073] | 0.096 [0.0948,0.0972] | 0.0005 [0.0004,0.0006] |
|  |  |  |  |  |
|  |  |  |  |  |
| **Mediation Analysis**  NDE  NIE | 0.020 [0.019,0.021]  0.0027 [0.0025,0.0029] | 0.008 [0.007,0.009]  0.0013 [0.0011,0.0015] | No mediator | 0.002 [-0.0003,0.0032]  -0.0007  [-0.0009, -0.0004] |
| **Double Machine Learning**  Linear  Non-parametric | 0.023 [0.0223,0.0236]  0.017 [0.0165,0.0175] | 0.009 [0.0087,0.0093]  0.006 [0.0057,0.0063] | 0.097 [0.096,0.098]  0.086 [0.0849,0.0871] | 0.002 [0.0018-0.0022]  0.000  [-0.0001,0.0001] |
| **Dragonnet** |  |  |  |  |
| Causal effect  Causal ratio | 0.017 [0.0169,0.0170]  2.44 [2.41,2.46] | 0.01 [0.009,0.010]  1.41 [1.21,1.60] | 0.075 [0.074,0.076]  4.56 [4.56,4.57] | -0.002 [-0.0022, -0.0021]  0.856 [0.855,0.858] |
|  |  |  |  |  |
| **Epidemiological Case-Control Study (OR)** | 2.56 [2.33,2.80] (28) | 1.16 [1.05,1.30] (28) | 3.34 [2.68,3.75]* (28) | 1.0[0.8,1.4] 1.6[1.3,1.9]** (29) |
|  |  |  |  |  |

* Heart disease

** top quintile low-density lipoprotein (LDL)
