# Supplementary figures and images for: Application of Dragonnet and Conformal Inference for Estimating Individualized Treatment Effects for Personalized Stroke Prevention: Retrospective Cohort Study
Source: JMIR Cardio. 2025 Jan 8;9:e50627. doi: 10.2196/50627 (PMC11735012; doi:10.2196/50627)

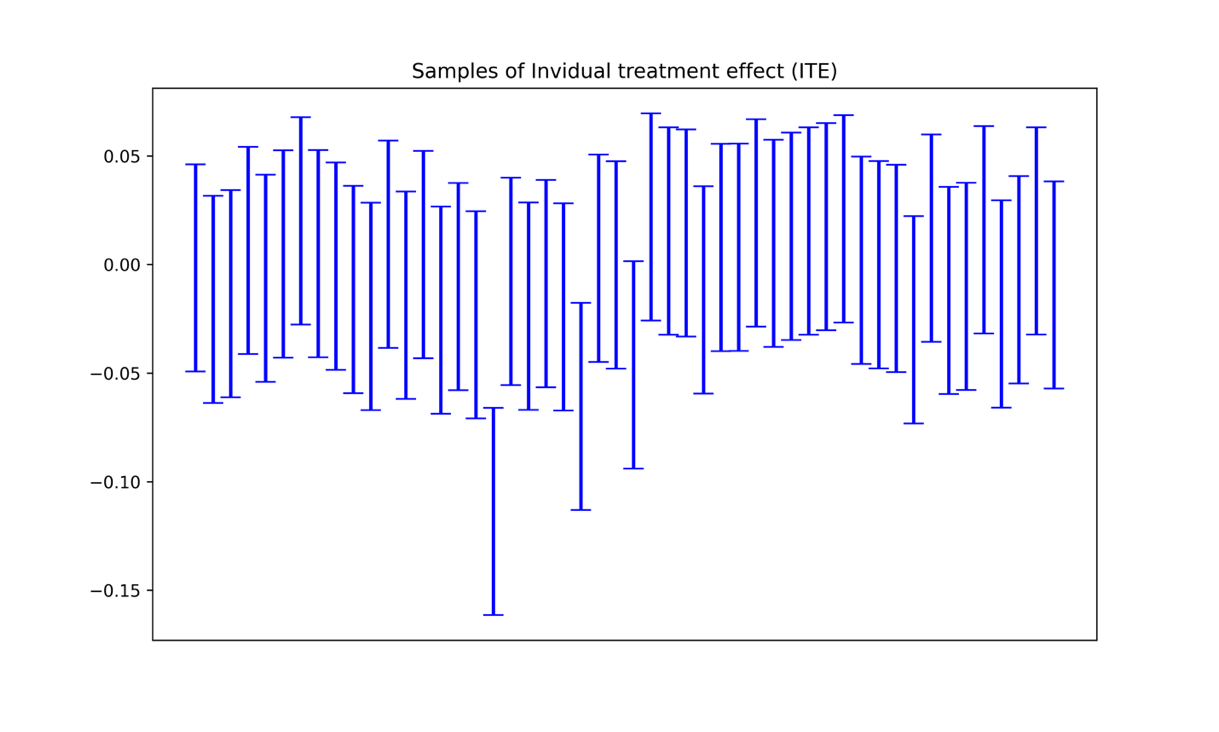

Supplement: Multimedia Appendix 4 [file cardio-v9-e50627-s004.docx]
